# Association between influenza vaccination and hospitalisation or all-cause mortality in people with COVID-19: a retrospective cohort study

**DOI:** 10.1136/bmjresp-2020-000857

**Published:** 2021-03-04

**Authors:** Christopher R Wilcox, Nazrul Islam, Hajira Dambha-Miller

**Affiliations:** 1 Primary Care Research Centre, University of Southampton, Southampton, UK; 2 Clinical Trial Service Unit and Epidemiological Studies Unit (CTSU), Nuffield Department of Population Health, University of Oxford, Oxford, Oxfordshire, UK

**Keywords:** COVID-19, respiratory infection, infection control, innate immunity

## Abstract

**Introduction:**

Recent evidence suggests that influenza vaccination may offer protection against COVID-19 severity. Our aim was to quantify the association between influenza vaccination status and risk of hospitalisation or all-cause mortality in people diagnosed with COVID-19.

**Methods:**

A retrospective cohort study using routinely collected health records from patients registered to a General Practitioner (GP) practice in South West England within the Electronic Care and Health Information Analytics database. The cohort included 6921 people with COVID-19 during the first wave of the pandemic (1 January–31 July 2020). Data on influenza vaccination, hospitalisation and all-cause mortality were ascertained through linked clinical and demographic records. We applied propensity score methods (stabilised inverse probability of treatment weight) to quantify the association between influenza vaccination status and COVID-19 outcomes (hospitalisation or all-cause mortality).

**Results:**

2613 (38%) participants received an influenza vaccination between 1 January 2019 and COVID-19 diagnosis. Receipt of influenza vaccination was associated with a significantly lower odds of hospitalisation or all-cause mortality (adjusted OR: 0.85, 95% CI 0.75 to 0.97, p=0.02), and 24% reduced odds of all-cause mortality (adjusted OR: 0.76, 95% CI 0.64 to 0.90).

**Discussion:**

Influenza vaccination was associated with a 15%–24% lower odds of severe COVID-19 outcomes. The current UK influenza vaccination programme needs urgent expansion as an integral component of the ongoing response plans to the COVID-19 pandemic.

**Key messages**

- We sought to determine the association between influenza vaccination status and risk of hospitalisation or all-cause mortality in people diagnosed with COVID-19.
- In people with COVID-19 during the first wave of the pandemic, we found that influenza vaccination was associated with a 15%–24% lower odds of hospitalisation or all-cause mortality.
- Influenza vaccination may have the potential to offer an additional means of mitigating serious adverse complications of COVID-19, and we therefore propose that a rapid expansion of the influenza vaccination programme is urgently needed as an integral component of the ongoing response to the COVID-19 pandemic.

## Background

COVID-19 caused by the SARS-CoV-2 is a rapidly evolving global public health emergency. Since the outbreak began, over 87 million cases have been reported globally, with over 1.8 million attributed deaths (as of 7 January 2020).^1^ Of those who develop symptoms, approximately 20% will have severe disease, including pneumonia and other life-threatening complications.^2^ Older adults and those with underlying heath conditions are particularly susceptible to severe outcomes; however, recent evidence suggests that even mild COVID-19 may result in prolonged symptoms and/or long-term adverse health outcomes.^3^ The pandemic has carried a substantial economic burden globally,^4^ and several countries have experienced unprecedented rise in demand on healthcare systems far exceeding capacity to respond.^5^

There remain limited effective interventions to treat COVID-19. A number of vaccines against SARS-CoV-2 have recently been approved for use following promising clinical trials^6 7^; however, ongoing challenges remain regarding the manufacturing, distribution and accessibility of vaccines internationally,^8^ and there is an ongoing drive to identify whether any available treatments could be repurposed to prevent COVID-19 acquisition and/or disease severity. One area of interest is in the role of existing vaccines. Vaccination against specific pathogens with both live-attenuated and inactivated vaccines can induce adaptive and innate immunity in the form of neutralising antibodies and/or antigen-specific cellular immunity. Growing evidence demonstrates that vaccination may also have a role in wider protection against unrelated pathogens.^9 10^ The underlying mechanism for this phenomenon remains poorly understood and may occur by induction of innate immune responses, so-called trained innate immunity.^9 11–14^ It is hypothesised that influenza vaccination may potentially have a direct protective effect against COVID-19.^6 9 15–17^ This is in addition to its potential indirect benefits, such as reducing the burden of additional respiratory illness among both individual patients and healthcare services.^18–20^

This hypothesis has been supported by a limited number of observational studies, including five retrospective cohort studies^15 17 21–23^ (three of which were limited by the use of aggregated data), demonstrating a negative association between influenza vaccination and COVID-19 outcomes including COVID-19 acquisition, severity and/or mortality. One prospective study using individual-level data demonstrated a negative association between influenza vaccination and COVID-19 acquisition but did not report on subsequent COVID-19 outcomes.^24^

Further validation is required from large UK-based cohorts using individual-level data, exploring important outcomes including all-cause mortality and hospitalisation. This may help us better understand the impact of influenza vaccination in COVID-19 disease and inform the current influenza vaccination programme during the second wave of the pandemic. The aim of this study was to quantify the association between influenza vaccination status and COVID-19 outcomes including hospitalisation and all-cause mortality.

## Methods

### Study design

Retrospective cohort analysis.

### Data source

Data were obtained from the Care and Health Information Analytics (CHIA) database. This is an anonymous National Health Service (NHS) database that includes individual-level data from 1.5 million patients across 160 GP practices in Southern England. Data have been extracted from routine general practice records linked to local secondary care hospitals, including demographic data, primary/secondary care consultations, diagnoses, medications and outcomes of primary/secondary care investigations.

### Study population

A cohort of individuals within the CHIA database diagnosed with COVID-19 during the first wave of the COVID-19 pandemic (1 January–31 July 2020) were identified. We included both confirmed and suspected cases. Confirmed cases had a positive RT-PCR assay for SARS-CoV-2 on nasal/pharyngeal swab. Clinically suspected cases were defined in accordance with Public Health England (PHE) guidance at the time of the first wave of the pandemic in the UK, when testing was not readily available. Further detail on the COVID-19 case definition has been reported in other databases,^25^ and our rationale for case definition can also be found in a consensus statement by the COVID-19 Primary Care Database Consortium.^26^ Recent evidence demonstrates that clinical suspicion of COVID-19 among GPs closely matches with confirmed cases and subsequent clinical outcomes.^27^

### Sociodemographic and clinical variables

Data for the identified cohort were extracted from the CHIA database including age, sex, ethnicity, Index of Multiple Deprivation (IMD), electronic frailty index, comorbidities, smoking status, body mass index (BMI), medications and influenza vaccination status.

### Exposure

Receipt of recent influenza vaccination was defined as an electronic recording of vaccination in the CHIA database between 1 January 2019 and the diagnosis of COVID-19.

### Outcomes

Since death is a competing risk for hospitalisation,^28^ our primary outcome was a composite of hospitalisation or all-cause mortality. Our secondary outcome was all-cause mortality alone. Incidence of hospitalisation and all-cause mortality was identified using linked hospital records and the Office for National Statistics. We did not analyse COVID-19 specific mortality (death within 28 days of diagnosis), as the exact dates of diagnosis were not sufficiently robust, and there was uncertainty about accuracy of electronic coding and certification of death nationally with regards to COVID-19 specific mortality in the first few months of the pandemic.

### Statistical analysis

Baseline demographics/clinical characteristics of the study cohort were summarised. Categorical variables were summarised as frequencies and percentages, and continuous variables were as means and SD.

Since our primary exposure variable, influenza vaccination, is based on observational data (as opposed to allocation by randomisation), the group of patients that received the vaccine is not directly comparable with the group that did not receive the vaccine. The reasons why some people received the vaccination leads to so-called treatment indication bias. We attempted to address this confounding by treatment indication bias within marginal structural regression framework, because conventional regression adjustment may not be sufficient to address this issue.^28 29^ Specifically, we estimated the comparative effectiveness of influenza vaccination by applying stabilised inverse probability of treatment weights (IPTW) for the receipt of influenza vaccination.^30^ Propensity score was estimated by fitting logistic regression using an a priori list of variables including age, sex, BMI, socioeconomic status (IMD), smoking status, frailty score (electronic frailty index), pre-existing comorbidities (chronic obstructive pulmonary disease, stroke, cancer, depression, peripheral arterial disease, rheumatoid arthritis, atrial fibrillation, dementia, chronic kidney disease, heart failure, learning disability, hypertension, other mental health disorder, cardiovascular disease, epilepsy, asthma, osteoporosis, coronary artery disease, osteoarthritis and diabetes) and the number of prescribed medications. The balance of the propensity score between the two vaccination groups was visually checked using histograms. The numerator for the stabilised IPTW was the marginal probability of vaccination receipt, estimated by fitting intercept-only logistic regression, for the group that received the influenza vaccine. The complement of this probability was the numerator for the group that did not receive the vaccination. Similarly, the propensity score was the denominator for the vaccinated group, while the complement of the propensity score was the denominator for the unvaccinated group.^31^ The effect estimates were reported as adjusted ORs and corresponding 95% CIs. Statistical analyses were undertaken using Stata SE V.15.1 (StataCorp, College Station, Texas, USA).

### Sensitivity analysis

To check the robustness of our analysis using stabilised IPTW, we estimated the pooled OR by stratifying the propensity score into deciles (10 equal groups). Mantel-Haenszel weighted pooled OR was estimated using Woolf approximation for the calculation of SE.^32^ In further sensitivity analysis, we defined influenza receipt only when it was administered within 6 months of COVID-19 pandemic in the UK (ie, when the vaccination was administered on or after 1 September 2019). Finally, we also performed an additional time-to-event analysis adjusting for month of COVID-19 diagnosis.

### Missing data

There was missing data on ethnicity, BMI, smoking, IMD and age. Ethnicity is frequently missing from routinely collected primary care records. We removed this variable from the analyses for multiple reasons: (1) high amount of missing data on ethnicity (32.8%), (2) very low number of outcome events in some subgroups (eg, there was one case of all-cause mortality in the Asian ethnic group) and (3) inadequate reduction of bias by traditional missing data imputation methods^32 33^ and (4) this geographical area of the UK has low proportion of ethnic minorities. For other variables with missing data, we used multiple imputation by chained equations assuming missing data were missing at random. We produced five sets of imputed datasets with 100 iterations in the burn-in period.^34 35^ The non-missing variables used to impute the missing variables were sex, frailty score and pre-existing comorbidities.

### Ethical considerations

CHIA is an anonymous NHS database, and all individuals have consented for collection of their medical records for inclusion in the database. Findings are reported as per Strengthening the Reporting of Observational Studies in Epidemiology and RECORD guidelines for observational studies of routinely collected data.

### Data availability

Anonymised individual level data used in this study was extracted from the CHIA database. Direct requests for data sharing can be made to CHIA.

### Patient and public involvement

Due to the nature of the study, patients or the public were not involved in the design, conduct, reporting or dissemination of the study

## Results

### Baseline characteristics

A total of 6921 participants were included in the study, of which 774 (11.2%) received a confirmed diagnosis of COVID-19 during the follow-up period, and 6147 (88.8%) were diagnosed clinically. The mean age was 52.4 years (±24.5) and the majority of participants (59.5%) were female. Most participants were white (60.2%); however, ethnicity was not recorded in one-third of cases (32.8%). Approximately half of participants were from the two least deprived socioeconomic status groups (32.8% and 22.9% from IMD groups 5 and 4, respectively). Baseline characteristics of the study cohort are summarised in table 1.

**Table 1:** Baselines characteristics of people with COVID-19, within the CHIA database cohort, stratified by influenza vaccination status

Characteristics	Influenza vaccination received		
	Total	Yes	No
N	6921	2613	4308
Age baseline, mean (SD)	52.4 (24.5)	61.7 (24.0)	46.7 (22.9)
Female, n (%)	4120 (59.5)	1577 (60.4)	2543 (59.0)
Ethnicity, n (%)			
Asian	328 (4.7)	98 (3.8)	230 (5.3)
Black	54 (0.8)	17 (0.7)	37 (0.9)
Missing data	2273 (32.8)	704 (26.9)	1569 (36.4)
Mixed or other	101 (1.5)	24 (0.9)	77 (1.8)
White	4165 (60.2)	1770 (67.7)	2395 (55.6)
Socioeconomic status (IMD), n (%)			
1 (most deprived)	617 (8.9)	244 (9.3)	373 (8.7)
2	1055 (15.2)	328 (12.6)	727 (16.9)
3	1207 (17.4)	477 (18.3)	730 (16.9)
4	1586 (22.9)	599 (22.9)	987 (22.9)
5 (least deprived)	2268 (32.8)	917 (35.1)	1351 (31.4)
Missing	188 (2.7)	48 (1.8)	140 (3.2)
Frailty score, mean (SD)	0.2 (0.2)	0.3 (0.2)	0.1 (0.1)
Frailty score, median (IQR)	0.1 (0.1–0.3)	0.2 (0.1–0.3)	0.1 (0.0–0.2)
Comorbidities, n (%)			
Hypertension	1396 (20.2)	864 (33.1)	532 (12.3)
Stroke	296 (4.3)	206 (7.9)	90 (2.1)
Asthma	966 (14.0)	576 (22.0)	390 (9.1)
Chronic obstructive pulmonary disease	334 (4.8)	246 (9.4)	88 (2.0)
Coronary heart disease	368 (5.3)	277 (10.6)	91 (2.1)
Heart failure	196 (2.8)	144 (5.5)	52 (1.2)
Type 1 diabetes	42 (0.6)	34 (1.3)	8 (0.2)
Type 2 diabetes	559 (8.1)	397 (15.2)	162 (3.8)
CKD stage 3–5	351 (5.1)	255 (9.8)	96 (2.2)
Smoking status, n (%)			
Current smoker	784 (11.3)	221 (8.5)	563 (13.1)
Ex-smoker	2171 (31.4)	974 (37.3)	1197 (27.8)
Never smoked	3279 (47.4)	1215 (46.5)	2064 (47.9)
Missing data	687 (9.9)	203 (7.8)	484 (11.2)
Body mass index	27.9 (6.9)	28.7 (7.4)	27.4 (6.5)
Medication, n (%)			
Antiplatelets	893 (12.9)	543 (20.8)	350 (8.1)
Oral anticoagulants	651 (9.4)	368 (14.1)	283 (6.6)
ACE inhibitors	977 (14.1)	554 (21.2)	423 (9.8)
Angiotensin receptor blockers	473 (6.8)	286 (10.9)	187 (4.3)
Diuretics	1051 (15.2)	627 (24.0)	424 (9.8)
Calcium channel blockers	957 (13.8)	567 (21.7)	390 (9.1)
Beta blockers	1117 (16.1)	567 (21.7)	550 (12.8)
Alpha blockers	141 (2.0)	95 (3.6)	46 (1.1)
Insulin	270 (3.9)	177 (6.8)	93 (2.2)
Diabetic oral agents	611 (8.8)	398 (15.2)	213 (4.9)
Lipid-lowering drugs including statins	1550 (22.4)	959 (36.7)	591 (13.7)

CHIA, Care and Health Information Analytics; CKD, chronic kidney disease; IMD, Index of Multiple Deprivation.

A total of 2613 (38%) participants in the cohort had received influenza vaccination between 1 January 2019 and COVID-19 diagnosis (see table 1). Compared with the unvaccinated people, those who received influenza vaccination on average were older (mean 61.7 years vs 46.7 years) and frailer (mean frailty score 0.3 vs 0.1).

### Influenza vaccination and outcomes

Baselines characteristics of the study cohort, stratified by incidence of the primary study outcome (hospitalisation or all-cause mortality), are displayed in table 2. Of the 2613 people who received an influenza vaccination, 372 (14.2%) died during the follow-up time and 1166 (44.6%) either died or were hospitalised. These proportions were 12.8% (n=553) and 36.8% (n=1584) in the 3755 people without vaccination. Those who died or had a hospital admission were on average older (mean 62.7 years vs 45.5), frailer (frailty score 0.2 vs 0.1) and a lower proportion were reported as white ethnicity (52.0% vs 65.6%), with more having missing ethnicity data (42.7% vs 26.3%).

**Table 2:** Baselines characteristics of people with COVID-19 within the CHIA database cohort, stratified by incidence of the study outcomes (hospitalisation or all-cause mortality)

Characteristics	Hospitalisation or all-cause mortality	
	Yes	No
N	2750	4171
Received seasonal influenza vaccination, n (%)	1166 (42.4)	1447 (34.7)
Age baseline; mean (SD)	62.7 (22.8)	45.5 (23.1)
Female, n (%)	1540 (56.0)	2580 (61.9)
Ethnicity, n (%)		
Asian	106 (3.9)	222 (5.3)
Black	17 (0.6)	37 (0.9)
Missing data	1174 (42.7)	1099 (26.3)
Mixed or other	23 (0.8)	78 (1.9)
White	1430 (52.0)	2735 (65.6)
Socioeconomic status (IMD), n (%)		
1 (most deprived)	280 (10.2)	337 (8.1)
2	479 (17.4)	576 (13.8)
3	539 (19.6)	668 (16.0)
4	611 (22.2)	975 (23.4)
5 (least deprived)	789 (28.7)	1479 (35.5)
Missing	52 (1.9)	136 (3.3)
Frailty score, mean (SD)	0.2 (0.2)	0.1 (0.2)
Frailty score, median (IQR)	0.2 (0.1–0.3)	0.1 (0.0–0.2)
Comorbidities, n (%)		
Hypertension	586 (21.3)	810 (19.4)
Stroke	154 (5.6)	142 (3.4)
Asthma	306 (11.1)	660 (15.8)
Chronic obstructive pulmonary disease	157 (5.7)	177 (4.2)
Coronary heart disease	176 (6.4)	192 (4.6)
Heart failure	115 (4.2)	81 (1.9)
Type 1 diabetes	15 (0.5)	27 (0.6)
Type 2 diabetes	256 (9.3)	303 (7.3)
CKD stage 3–5	173 (6.3)	178 (4.3)
Smoking status, n (%)		
Current smoker	291 (10.6)	493 (11.8)
Ex-smoker	1027 (37.3)	1144 (27.4)
Never smoked	1292 (47.0)	1987 (47.6)
Missing data	140 (5.1)	547 (13.1)
Body mass index	28.0 (7.0)	27.8 (6.8)
Medication, n (%)		
Antiplatelets	561 (20.4)	332 (8.0)
Oral anticoagulants	437 (15.9)	214 (5.1)
ACE inhibitors	539 (19.6)	438 (10.5)
ARB	236 (8.6)	237 (5.7)
Diuretics	681 (24.8)	370 (8.9)
Calcium channel blockers	547 (19.9)	410 (9.8)
Beta blockers	656 (23.9)	461 (11.1)
Alpha blockers	87 (3.2)	54 (1.3)
Insulin	179 (6.5)	91 (2.2)
Diabetic oral agents	363 (13.2)	248 (5.9)
Lipid-lowering drugs including statins	894 (32.5)	656 (15.7)

CHIA, Care and Health Information Analytics; CKD, chronic kidney disease; IMD, Index of Multiple Deprivation.

**Figure 1:**
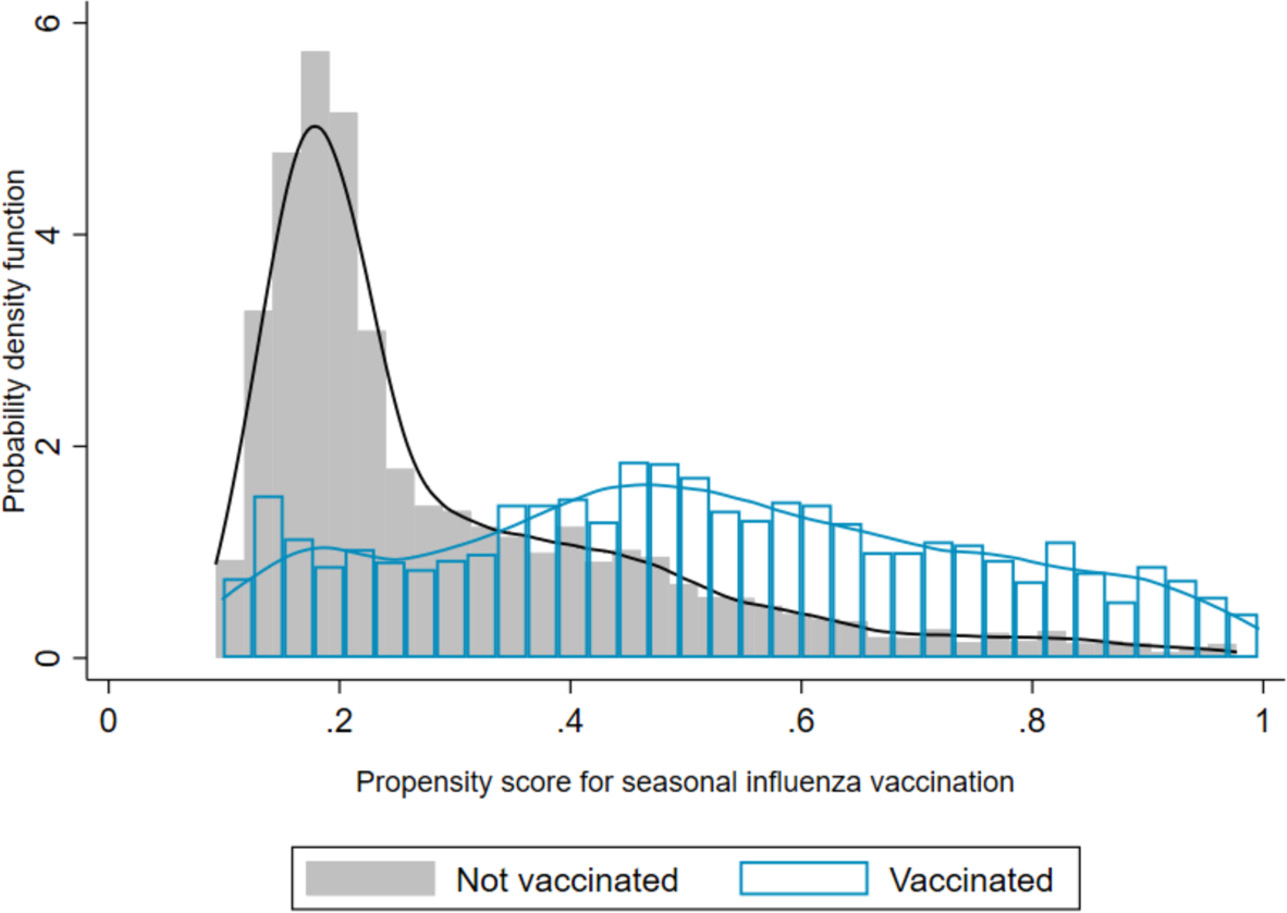
Distribution of the propensity score for patients who received seasonal influenza vaccination (n=2613) compared with those who did not receive the vaccination (n=4308) since January 2019.

The overall distribution of the propensity score by influenza vaccination status is shown in figure 1 while the mean (SD) of the propensity score by deciles of the propensity score is shown in online supplementary table S1. The distribution shows a fairly strong common support and balanced distribution in the two vaccination groups.

10.1136/bmjresp-2020-000857.supp1 Supplementary data

In the stabilised IPTW-weighted regression model, influenza vaccination was associated with 15% reduced odds of hospitalisation or all-cause mortality (adjusted OR: 0.85, 95% CI 0.75 to 0.97; p=0.02) and 24% reduced odds of all-cause mortality (adjusted OR: 0.76, 95% CI 0.64 to 0.90) (see table 3). Furthermore, on performing additional time-to-event analysis, we found that the results were identical to our primary analysis when we additionally adjusted for the month of COVID-19 diagnosis.

**Table 3:** Association between influenza vaccination and hospitalisation or all-cause mortality, and all-cause mortality alone, in people with COVID-19 within the CHIA database cohort (n=6921)

Outcome	Model specification	OR (95% CI)	P value
Hospitalisation or all-cause mortality	Unadjusted	1.39 (1.26 to 1.53)	<0.001
	Stabilised IPTW*	0.85 (0.75 to 0.97)	0.020
	Stratification and pooling	0.87 (0.78 to 0.98)	0.021
All-cause mortality	Unadjusted	1.13 (0.98 to 1.30)	0.097
	Stabilised IPTW†	0.76 (0.64 to 0.90)	0.001
	Stratification and pooling	0.59 (0.51 to 0.69)	<0.001

*IPTW: inverse probability of treatment weighting based on the propensity score for influenza vaccination stabilised by the marginal probability of vaccination in the study population.

†The propensity score was divided into deciles, and the OR for the association between influenza vaccination and the outcome was estimated in each of the deciles. Mantel-Haenszel-weighted pooled OR was estimated using Woolf approximation for the calculation of SE.

CHIA, Care and Health Information Analytics.

In the sensitivity analysis using pooled OR after stratification by deciles of propensity score, the results were almost identical for the primary outcome (adjusted OR: 0.87, 95% CI 0.78 to 0.98, p=0.021) with a slightly stronger effect for all-cause mortality (adjusted OR: 0.59, 95% CI 0.51 to 0.69, p<0.001) (see table 3). In the sensitivity analysis with a modified definition of influenza vaccination (administered on or after 1 September 2020), the effects were almost identical to our primary analysis (adjusted OR: 0.85, 95% CI 0.74 to 0.97, p=0.016 for hospitalisation or all-cause mortality, and 0.78, 95% CI 0.66 to 0.92, p=0.004 for all-cause mortality).

## Discussion

### Key findings

In this sample of 6921 participants who received a diagnosis of COVID-19 during the first wave of the pandemic (1 January–31 July 2019), we found that having prior influenza vaccination was associated with a 15%–24% lower odds of hospitalisation or all-cause mortality.

### Comparison with existing literature

Our study is consistent with observational data from outside the UK demonstrating a negative association between influenza vaccination and COVID-19 acquisition, severity and mortality.^12 14 18 35 36^ An recent Italian retrospective study demonstrated a modest negative correlation between the percentage of adults aged >65 years who had received influenza vaccination and the percentage of COVID-19 deaths from each region of Italy (r: −0.59).^15^ Similarly, a US cohort of adults aged ≥65 years demonstrated that a 10% increase in vaccination uptake was associated, on average, with a 28% decrease in the COVID-19 mortality rate.^22^ Another study examined influenza vaccination data from 34 countries (using Organisation for Economic Cooperation and Development data) with COVID-19 mortality from worldometer data and reported a negative correlation between influenza vaccination status and COVID-19 mortality (R^2^ 0.338).^17^ These studies were limited by the use of aggregated data, however. Two retrospective studies using patient-level data include a large Italian cohort study in which receipt of influenza vaccination was associated with 11% lower odds of COVID-19 diagnosis, and among a subgroup of those aged ≥65 years vaccinated in the first half of the vaccination programme, a 44% and 30% lower odds of hospitalisation and mortality, respectively. Another large retrospective cohort study in Brazil of 53 752 hospitalised patients with COVID-19 found that receipt of recent influenza vaccination was associated with 7%, 17% and 16% lower odds of intensive care admission, requiring invasive respiratory support and mortality, respectively. Finally, there is one prospective observational study in the USA that includes individual patient level data through a registry of 11 672 people tested for COVID-19 (of whom, 7% tested positive).^24^ It reports that influenza vaccination was associated with a lower risk of acquiring COVID-19 (p<0.001, OR not reported) but does not report on subsequent COVID-19 outcomes, including mortality.

Possible explanations for these findings include a direct protective effect against COVID-19 from influenza vaccination. Growing evidence, particularly from work studying BCG and measles vaccination,^9 11 12^ as well as more recently from influenza vaccination,^13 14^ demonstrates that vaccination may have a role in wider protection against unrelated pathogens, in addition to inducing adaptive/innate immunity against specific pathogens. The underlying mechanisms for these non-specific effects remain poorly understood. One suggested mechanism is the induction of innate immune responses (including monocytes and natural killer cells), so-called trained innate immunity, following live attenuated vaccination, in a way that is independent of memory T or B cells.^9 11–14^ This trained immunity may confer non-specific protection against different pathogens, inducing upregulation of pattern recognition receptors and the secretion of proinflammatory cytokines through epigenetic and metabolic reprogramming.^11–14^ Another proposed mechanism is a process called ‘emergency granulopoiesis’.^37^ This is based on the recent observation that following BCG vaccination in newborns; for example, there is induction of the growth factor granulocyte colony-stimulating factor which, in turn, leads to the rapid production of neutrophils, resulting in additional protection from subsequent infection.^38^ Finally, influenza vaccination may also offer additional indirect protection against COVID-19 by reducing the burden of additional respiratory illness among both individual patients and healthcare services.^18–20^

### Strengths and limitations

To our knowledge, this is the first study to examine influenza vaccination in relation to all-cause mortality and hospitalisation based on individual-patient data linked to demographic and clinical records in the UK. We applied robust analytic techniques to reduce the treatment indication bias that is inherent to observational studies. However, this does not completely rule out the possibility of residual and unknown confounding. A strength of the study is that it was drawn from a large database of individuals 160 GP practices across South West England; however, the cohort does include low representation of ethnic minority groups, and a relatively large proportion were from high socioeconomic backgrounds. Another limitation is our inability to analyse the effects by ethnicity due to large amount of missing data. Data being from a centralised single-payer healthcare system may limit the generalisability of these findings to other parts of the world.

The case definition of COVID-19 disease has also evolved over time, especially as testing has become more readily available in recent months. We included clinically suspected cases of COVID-19 alongside confirmed cases, in accordance with guidance from PHE during the first wave of the pandemic. Recent studies have shown that clinical suspicion of COVID-19 among GPs does closely match with confirmed cases and subsequent clinical outcomes, but it is still plausible that not all those with clinical cases had COVID-19.^27^ The clinical condition of patients at the time of diagnosis is also likely to have varied over the course of the study period (again as testing has become more readily available), as has the clinical management of patients, as clinicians have become more familiar with the COVID-19 over time. An additional time-to-event analysis in which we adjusted for the month of COVID-19 diagnosis showed identical results to our primary analysis, however. Our sample was also restricted to individuals with complete consultation notes over the study period. It is possible that individuals with incomplete records, mild symptoms only (or were asymptomatic) or attended hospital directly without first attending general practice may be under-represented in our sample. There is also a well-recognised ‘frailty bias’,^39^ whereby as people get older and frailer, vaccination rates tends to drop, and it is therefore plausible that this could have influenced our findings. However, we tried to take this into consideration by including frailty index, age and comorbidities within the regression model. Finally, the type of influenza vaccination given was not recorded in the database, and we are therefore unable to explore whether there is a difference between inactivated and live-attenuated vaccination on COVID-19 outcomes.

We used logistic regression for our primary analysis because the date of COVID-19 diagnosis, hospitalisation or deaths was not precise in the database. However, as stated above, additional time-to-event analysis adjusted for the month of COVID-19 diagnosis showed identical results. Furthermore, exact date of death was not always available for patients who died (only months and years of death were reported). Since the follow-up time was small, this is unlikely to have changed the estimates substantially.^40^ To verify this, we imputed the date as the 15th of the respective months and ran additionally sensitivity analyses on each of the outcomes using stabilised IPTW-weighted Cox’s proportional hazards model to estimate the adjusted HR and 95% CI. This robustly supported our findings; adjusted HR for the primary outcome (hospitalisation or all-cause mortality) and the secondary outcome (all-cause mortality) was 0.88 (95% CI 0.79 to 0.98, p=0.024) and 0.79 (95% CI 0.66 to 0.94, p=0.008), respectively.

### Implications of these findings

Our results suggest that influenza vaccination may have the potential to offer an additional means of mitigating serious adverse complications of COVID-19. Further research should aim to validate these findings in larger more diverse cohorts. Additional work is also needed to examine differences between influenza vaccine subtypes and whether the duration from vaccination administration to COVID-19 acquisition is important.

## Conclusion

In people with COVID-19 during the first wave of the pandemic, we found that influenza vaccination was associated with a 15%–24% lower odds of hospitalisation or all-cause mortality. An expansion of the influenza vaccination programme is needed as an integral component of the ongoing response to the COVID-19 pandemic.

## Data Availability

Data are available on reasonable request. Anonymised individual level data used in this study was extracted from the Care and Health Information Analytics (CHIA) database. Direct requests for data sharing can be made to CHIA.
