# Correction: Association between influenza vaccination and hospitalisation or all-cause mortality in people with COVID-19: a retrospective cohort study

**DOI:** 10.1136/bmjresp-2020-000857corr1

**Published:** 2021-04-07

Wilcox CR, Islam N, Dambha-Miller H. Association between influenza vaccination and hospitalisation or all-cause mortality in people with COVID-19: a retrospective cohort study. *BMJ Open Resp Res* 2021;8:e000857.

The authors want to alert readers to the following correction made to the published version.

Christopher Wilcox and Nazrul Islam are the joint first authors.

