# Supplementary material for: Association between influenza vaccination and hospitalisation or all-cause mortality in people with COVID-19: a retrospective cohort study
Source: BMJ Open Respir Res. 2021 Mar 4;8(1):e000857. doi: 10.1136/bmjresp-2020-000857 (PMC7934200; doi:10.1136/bmjresp-2020-000857)
Supplement: Supplementary data [file bmjresp-2020-000857supp001.pdf]

Supplementary Table S1: Distribution of propensity scores (by deciles) stratified by receipt of seasonal influenza vaccination

	Receipt of influenza vaccination	
**Deciles of propensity score**	**Yes**	**No**
1	0.13 (0.01)	0.13 (0.01)
2	0.16 (0.01)	0.16 (0.01)
3	0.19 (0.01)	0.19 (0.01)
4	0.22 (0.01)	0.22 (0.01)
5	0.28 (0.02)	0.27 (0.02)
6	0.36 (0.02)	0.35 (0.02)
7	0.44 (0.02)	0.43 (0.02)
8	0.52 (0.03)	0.52 (0.03)
9	0.65 (0.05)	0.64 (0.05)
10	0.85 (0.07)	0.84 (0.07)

- Data are shown in mean (SD).
